# An equity evaluation in stroke inpatients in regard to medical costs in China: a nationwide study

**DOI:** 10.1186/s12913-021-06436-x

**Published:** 2021-05-05

**Authors:** Yong Yang, Stephen Nicholas, Elizabeth Maitland, Zhengwei Huang, Xiaoping Chen, Yong Ma, Xuefeng Shi

**Affiliations:** 1 School of Management, Beijing University of Chinese Medicine, No. 11, Bei San Huan Dong Lu, Chaoyang District Beijing, 100029 People’s Republic of China; 2 Medical Device Regulatory Research and Evaluation Center, West China Hospital, Sichuan University, Chengdu, China; 3 Australian National Institute of Management and Commerce, 1 Central Avenue Australian Technology Park, Eveleigh Sydney, NSW 2015 Australia; 4 School of Economics and School of Management, Tianjin Normal University, Tianjin, China; 5 Guangdong Institute for International Strategies, Guangdong University of Foreign Studies, Guangzhou, China; 6 Newcastle Business School, University of Newcastle, Newcastle, Australia; 7 University of Liverpool Management School, University of Liverpool, Liverpool, L697ZH UK; 8 China Health Insurance Research Association, Beijing, China; 9 National Institute of Traditional Chinese Medicine Strategy and Development, Beijing University of Chinese Medicine, Beijing, China

**Keywords:** Stroke, Equity, Health policy, Health economics, Theil index

## Abstract

**Background:**

Stroke has always been a severe disease and imposed heavy financial burden on the health system. Equity in patients in regard to healthcare utilization and medical costs are recognized as a significant factor influencing medical quality and health system responsiveness. The aim of this study is to understand the equity in stroke patients concerning medical costs and healthcare utilization, as well as identify potential factors contributing to geographic variation in stroke patients’ healthcare utilization and costs.

**Methods:**

Covering 31 provinces in mainland China, our main data were a 5% random sample of stroke claims from Urban Employees Basic Medical Insurance (UEBMI) and Urban Residents Basic Medical Insurance (URBMI) from 2013 to 2016. The Theil index was employed to evaluate the equity in stroke patients in regard to healthcare utilization and medical costs, and the random-effect panel model was used to explore the impact of province-level factors (health resource factors, enabling factors, and economic factors) on medical costs and health care utilization.

**Results:**

Stroke patients’ healthcare utilization and medical costs showed significant differences both within and between regions. The UEBMI scheme had an overall lower Theil index value than the URBMI scheme. The intra-region Theil index value was higher than the inter-region Theil index, with the Theil index highest within eastern China, China’s richest and most developed region. Health resource factors and enabling factors (represented by reimbursement rate and education attainment years) were identified significantly associated with medical costs (*P* < 0.05), but have no impact on average length of stay.

**Conclusions:**

China’s fragmented urban health insurance schemes require further reform to ensure better equity in healthcare utilization and medical costs for stroke patients. Improving education attainment, offering equal access to healthcare, allocating health resources reasonably and balancing health services prices in different regions also count.

## Background

With characteristics of high morbidity, high recurrence rate, high disability rate and high mortality, stroke remains the second leading cause of death and a major cause of disability worldwide, imposing a heavy financial burden to health system [1]. It was reported that western countries spent approximately 3 to 4% of total health care expenditures on stroke [2]. And in China, the newest evidence showed that nearly 2% of total health expenditures of urban residents were spent on stroke-related medical costs [3]. Previous studies have assessed medical costs for stroke patients in some specific cities in China: In Guangzhou, the average medical costs was $3212.1 from 2006 to 2013 [4], and it was calculated as $3052.7 in Beijing city in 2012 [5]. It seemed that geographic variation in stroke patients’ medical costs widely existed in China. While we know little about the level of fairness/unfairness on stroke patients’ medical costs.

As a common pursuit of human society, equity has attracted increasing attention from the public, and was considered as one of the main issues in the healthcare system [6, 7]. Equity in patients in regard to healthcare utilization and medical costs has always been recognized as significant factors influencing medical quality and health system responsiveness [8]. Improved equity in medical costs and healthcare utilization can contribute to mitigation of disparities in health care access for stroke patients from different regions. In order to promote equity in healthcare, the Chinese government has established a large and well-functioning social health insurance system, including two insurance schemes designed exclusively for urban residents: Urban Employee Basic Medical Insurance scheme (UEBMI) for the urban employed and the Urban Resident Basic Medical Insurance scheme (URBMI) for primary and secondary school students, children and other urban unemployed residents [9]. The two schemes covered roughly 750 million, or 54%, of the total Chinese population in 2016 [10]. The healthcare system was considered to have made substantial progress in improving equal access to care and strengthening financial protection in the past few decades [11]. The major differences between UEBMI and URBMI lie in source of funding, reimbursement rate, reimbursement ceiling and benefit packages. Since UEBMI is funded both by employers (2% of wages) and employees (6% of annual wages) [12], the revenue for URBMI is from individual premium contributions and subsides from central and local government. In 2016, the per-capita fund of UEBMI was $523.7, and $94.3, for URBMI. Usually, patients with UEBMI enjoy a higher reimbursement rate than the URBMI patients, which means UEBMI provides better financial protection than URBMI. In addition, UEBMI offers a more comprehensive coverage than URBMI, which focuses on inpatient services and catastrophic illness, but with limited coverage of basic outpatient services [13]. In addition, the reimbursement ceiling of a UEBMI patient was set as six times of local employees’ annual average wage per year, while six times of local household disposable income per year for a URBMI patient.

After implementation plans from provincial governments, UEBIM and URBMI were risk pooled across 333 municipalities (Beijing, Chongqing, Tianjin, Shanghai) and prefectures (administrative units within a province), which implies that there are approximately 333 different UEBMI schemes and URBMI schemes in the country [14]. Disparities exist in the benefit package, reimbursement rate and reimbursement ceiling within each local UEBMI and URBMI scheme [14]. Previous studies have identified that the UEBMI beneficiaries utilized more health services and had higher medical costs than those with URBMI [3, 4]. But we still don’t know whether there were disparities in equity in patients’ medical costs and healthcare utilization between UEBMI and URBMI in major diseases such as stroke. Have UEBMI stroke patients been experiencing greater equity than URBMI patients?

The equity in patients in regard to health expenditures has attracted the interests of many scholars in the field of public health. Various methods were adopted to conduct the research on equity in patients in regard to health expenditure, such as concentration index and Lorenz curve [15], Gini-coefficient [16], Theil index [17–19] and so on. Especially the Theil index, which was a useful tool used worldwide to measure equity, not only in China, but also in Ethiopia [18], India [20], and Bangladesh [21]. In this study, we used Theil index to measure the equity in the UEBMI and URBMI stroke patients in regard to medical costs and healthcare utilization, as well as to compare which insurance scheme was fairer. Further, we discussed the provincial-level factors influencing the geographical variation of stroke patients’ healthcare utilization and medical costs. Our findings are expected to provide possible advice for promoting equity in stroke patients’ medical costs, and most importantly, to provide new evidence for optimizing China’s fragment social health insurances schemes.

## Methods

### Data resources and regional division

We collected a 5% random sample of UEBMI and URBMI insured stoke inpatients’ claim data between January 1, 2013 to December 31, 2016. And supporting data were from the China Labor Statistics Yearbook 2014–2017, the China Health Statistics Yearbook 2014–2017 and the China Statistical Yearbook 2014–2017. Inpatients data were covered by all 31 provinces, and municipalities of mainland China, excluding Macao, Hong Kong and Taiwan. Based on their gross domestic product (GDP) per capita, the 31 provinces were categorized into three groups, the eastern, central and western regions. The eastern region, including Beijing, Tianjin, Hebei, Liaoning, Shanghai, Jiangsu, Zhejiang, Shandong, Guangdong, and Hainan, had, on average, the highest average GDP per capita; the central region, including Shanxi, Jilin, Heilongjiang, Anhui, Jiangxi, Henan, Hubei and Hunan, had the next highest average per capital GDP; and the western region, including Inner Mongolia, Guangxi, Chongqing, Sichuan, Guizhou, Yunnan, Tibet, Shanxi, Gansu, Qinghai, Ningxia, Xinjiang, had the lowest average per capita GDP.

### Measuring tool

The Theil index is a relative indicator measure of economic and other types of inequality [22]. The advantage of the Theil index is that it can calculate the contribution of intra-group and inter-group inequities to total inequities, thus avoiding the calculation of absolute values. The Theil index ranges between 0 and 1, where smaller values point to more equitable distribution of some economic phenomena, such as income, across a population [23]. While originally used to measure inequalities in economic data, the Theil index is increasingly used to evaluate (in) equities in health services, including healthcare utilization and health service expenses [18]. The Theil index formula is:

1 $$ T=\sum \limits_{i=1}^N{p}_i{r}_i\ln \left({r}_i\right) $$

where P_i_ is the proportion of the population of province i, r_i_ is the ratio of the health indicator prevalence in province i to the overall health indicator prevalence in the population. While the Theil index has another form to measure the inequality between different groups [24]. Since we divided the 31 provinces into three regions, the Theil index in formula (1) can also be decomposed into the T_intra_, which measures utilization/expenditure inequality “within region”

2 $$ {T}_{intra}=\sum \limits_{i=1}^k{P}_g{T}_g $$

where P_g_ is the proportion of insured population in one region accounting for the total insured population and T_g_ is the Theil index of one region (eastern, central and western China). The Theil index (1) can also be decomposed into T_inter_, which measures utilization/expenditure inequality “between regions”.

3 $$ {T}_{inter}=\sum \limits_{i=1}^k{P}_g\mathit{\log}\frac{P_g}{Y_g} $$

where Y_g_ is the proportion of healthcare utilization/expense in one region accounting for the total healthcare utilization/expense. Higher T_intra_ and T_inter_ index values mean greater unfairness, while lower index values mean more equality.

4 $$ T={T}_{intra}+{T}_{inter} $$

The total Theil index value which reflects the level of equity in stroke patients in regard to medical costs/healthcare utilization in the whole country equals to the sum of T_intra_ and T_inter_. Similarly, higher T index values mean greater inequity and vice versa.

### Main indicators and statistical analysis

The Theil index was calculated with two indicators of expenses, medical costs (average direct medical costs for stroke patients in one province in one year) and OOP expenses (average share of medical costs paid by stroke patients in one province in one year), and one indicator of healthcare utilization, measured as average length of stay (ALOS). Based on the study framework of Anderson’s healthcare utilization model [19], results of previous studies and data availability, we chose health resource factors, enabling factors facilitating healthcare-seeking, and economic factors to explore the potential factors influencing geographic variation of stroke patients’ healthcare utilization and medical costs [25–28]. The independent variables and dependent variables are all at provincial level. In our study, the health resource factors contain two aspects: the number of healthcare staff per 1000 residents (Staff) and the number of actual open beds per 1000 residents (Beds). These data were obtained from the China Health Statistics Yearbook 2014–2017. The enabling factors facilitating or impeding the use of healthcare services comprised the regional reimbursement rate, regional average education attainment years and regional urban employment rate (the proportion of urban workers accounting for all the urban residents in a province). The economic factors were represented by the insurance fund per capita, measured by the total health insurance revenue divided by the insured population in that year, and GDP per capita. These data were collected from the China Labor Statistics Yearbook 2014–2017 and the China Statistical Yearbook 2014–2017. The average annual RMB-US exchange rate was used from 2013 to 2016, which was US$0.159.

Descriptive statistics were employed to illustrate the regional distribution of medical costs and healthcare utilization for stroke patients. After conducting Hausman test, the influencing factors were identified using panel time-series model with a random effect. Statistical analyses were conducted using STATA version 14.0 (Stata Corp LP, College Station, TX), with statistical significance α = 0.05.

## Results

### Regional distribution of medical costs and utilization for stroke inpatients in 2016

To assess equity in the regional distribution of medical costs and healthcare utilization for stroke patients, we made a detail calculation of medical costs, OOP expenses, and ALOS in each of the 31 provinces and municipalities and in each of the three regions. As shown in Table 1, there were significant differences in patients’ healthcare utilization and expenditure between regions, within regions and between provinces. For example, within the eastern region, Beijing had higher UEBMI medical costs (RMB21793.1/$3465.1) than all other eastern provinces, except Zhejiang (RMB26955.3/$4285.8); higher OOP expenses (RMB4611.8/$733.3) except for Tianjin (RMB5439.9/$864.9); and higher ALOS (16.2 days) except Zhejiang (27.8 days), Shanghai (26.5 days) and Guangdong (18.7 days). Within the central region, Hunan had the highest UEBMI medical costs (RMB 23804.2/$3784.9), OOP expenses (RMB 6111.3/$971.7) and URBMI OOP expenses (RMB5915.8/$940.6) than any other central provinces. Jiangxi had the highest URBMI medical costs (RMB12074.4/$1919.8) among central provinces. Within western China, in the UEBMI group, Qinghai had the highest medical costs (RMB 18432.0/$2930.7) and in Xinjiang, it was the lowest (RMB 7629.2/$1213.0). Guizhou had the lowest OOP expenses (RMB1584.2/$251.9 but the longest ALOS (20.3 days) among western provinces. While in the URBMI group, Xizang had the highest medical costs (RMB 27833.0/$4425.4) and OOP expenses (RMB10479.3/$1666) than all other western provinces.

**Table 1:** Regional distribution of healthcare utilization for UEBMI and URBMI stroke inpatients in 2016

Region	Provinces	UEBMI			URBMI		
		Medical costs (RMB)	OOP expenses (RMB)	ALOS (days)	Medical costs (RMB)	OOP expenses (RMB)	ALOS (days)
East	Beijing	21,793.1	4611.8	16.2	18,848.4	7568.8	14.5
	Tianjin	15,955.8	5439.9	12.4	10,397.5	5558.6	10.2
	Hebei	17,740.5	4054.9	15.8	13,863.8	5967.1	13.5
	Liaoning	11,188.0	2395.7	12.8	8304.6	3081.0	11.1
	Jiangsu	14,754.0	3279.4	15.2	11,356.7	3897.6	12.0
	Zhejiang	26,955.3	2546.3	27.8	15,190.4	4686.9	15.7
	Shandong	11,751.7	3201.4	15.1	8522.1	3746.8	11.3
	Guangdong	19,946.9	4960.5	18.7	15,452.9	11,588.9	15.1
	Hainan	13,806.0	2224.3	13.8	11,884.1	4397.9	12.4
	Fujian	18,506.2	4661.9	17.9	16,138.3	7964.8	14.9
	Shanghai	17,910.3	3602.3	26.5	16,003.4	3901.6	33.3
	***P*-value**	**< 0.001**	**< 0.001**	**< 0.001**	**< 0.001**	**< 0.001**	**< 0.001**
Central	Shanxi	12,249.3	2758.5	14.6	10,368.9	3883.6	15.1
	Jilin	10,967.3	3202.6	15.3	9030.7	4320.0	13.4
	Hei Longjiang	10,254.3	3099.2	12.4	9578.2	5586.3	12.5
	Anhui	11,947.9	3136.4	13.9	11,549.3	5595.4	13.2
	Jiangxi	13,725.0	3057.1	16.5	12,074.4	5401.3	13.2
	Henan	10,160.8	2279.3	14.1	7902.8	3379.3	12.3
	Hubei	9639.3	2211.5	13.9	6117.3	2450.1	11.4
	Hunan	23,804.2	6111.3	13.1	11,107.8	5915.8	11.7
	***P*-value**	**< 0.001**	**< 0.001**	**< 0.001**	**< 0.001**	**< 0.001**	**< 0.001**
West	Inner Mongolia	17,933.0	5075.6	15.8	19,327.1	8833.4	13.4
	Guangxi	13,733.7	2672.8	17.2	11,339.2	4366.5	13.7
	Chongqing	14,982.6	3975.1	15.1	8178.7	4064.7	10.1
	Sichuan	14,861.2	3325.2	17.8	9643.0	3742.4	12.9
	Guizhou	11,769.3	1584.2	20.3	9825.7	4870.6	18.7
	Yunnan	12,257.1	2217.1	16.0	8482.5	3330.1	11.4
	Xizang	17,908.5	2059.6	15.3	27,833.0	10,479.3	17.6
	Shanxi	14,111.3	3914.5	13.1	10,703.4	5725.9	10.5
	Gansu	8877.6	2069.1	13.0	10,378.2	4996.8	12.3
	Qinghai	18,432.0	4415.5	16.4	14,656.0	6906.7	20.3
	Ningxia	12,412.9	3711.1	16.0	7444.2	2559.6	12.2
	Xinjiang	7629.2	1812.5	12.2	5776.6	2488.3	10.4
	***P*-value**	**< 0.001**	**< 0.001**	**< 0.001**	**< 0.001**	**< 0.001**	**< 0.001**
***P*-value (between regions)**		**< 0.001**	**< 0.001**	**< 0.001**	**< 0.001**	**< 0.001**	**< 0.001**
***P*-value (between provinces)**		**< 0.001**	**< 0.001**	**< 0.001**	**< 0.001**	**< 0.001**	**< 0.001**

UEBMI Urban Employee Basic Medical Insurance scheme, URBMI Urban Resident Basic Medical Insurance scheme, ALOS average length of stay, OOP out-of-pocket; all *P*-values were based on Kruskal-Wallis test

Within region hospital expenses and utilization rates in Table 1 point to significant inequalities in healthcare within region, between regions and between provinces.

### Theil index of healthcare utilization and medical costs for stroke inpatients

The Theil index values in Table 2 show that there were significant variations in medical costs, OOP expenses and ALOS under both UEBMI and URBMI. As shown in Table 2, the year-by-year UEBMI Theil index of hospital cost fluctuated significantly, from 0.1256 in 2013 to 0.1700 in 2014, then back to 0.1214 in 2016. While the URBMI hospital cost index fluctuated up and down year-by-year, URBMI ALOS index rose every year. To further capture changes in the Theil index between UEBMI and URBMI, Table 2 calculates the difference of the Theil index values between UEBMI and URBMI, which shows that with higher numbers of Theil index values, the URBMI inpatients suffered greater unfairness in medical cost and health care utilization than the UEBMI inpatients. Second, the differences in Table 2 also show that these differences varied significantly year-by-year.

**Table 2:** Theil index of healthcare utilization and medical costs for stroke inpatients from 2013 to 2016

Year	UEBMI			URBMI			D_1_	D_2_	D_3_
	Medical costs^①^	OOP expenses^②^	ALOS^③^	Medical costs^④^	OOP expenses^⑤^	ALOS^⑥^			
2013	0.1256	0.1238	0.1397	0.2651	0.2208	0.1918	0.1395	0.0970	0.0521
2014	0.1700	0.1998	0.1388	0.2061	0.1756	0.2090	0.0361	−0.0243	0.0702
2015	0.1433	0.1585	0.1424	0.2794	0.3322	0.2134	0.1361	0.1737	0.0710
2016	0.1214	0.0929	0.1444	0.2352	0.2401	0.2371	0.1038	0.1473	0.0927

UEBMI Urban Employee Basic Medical Insurance scheme, URBMI Urban Resident Basic Medical Insurance scheme; ALOS average length of stay, OOP out-of-pocket; D_1_ = group④-group①; D_2_ = group⑤-group②; D_3_ = group⑥-group③

**Fig. 1:**
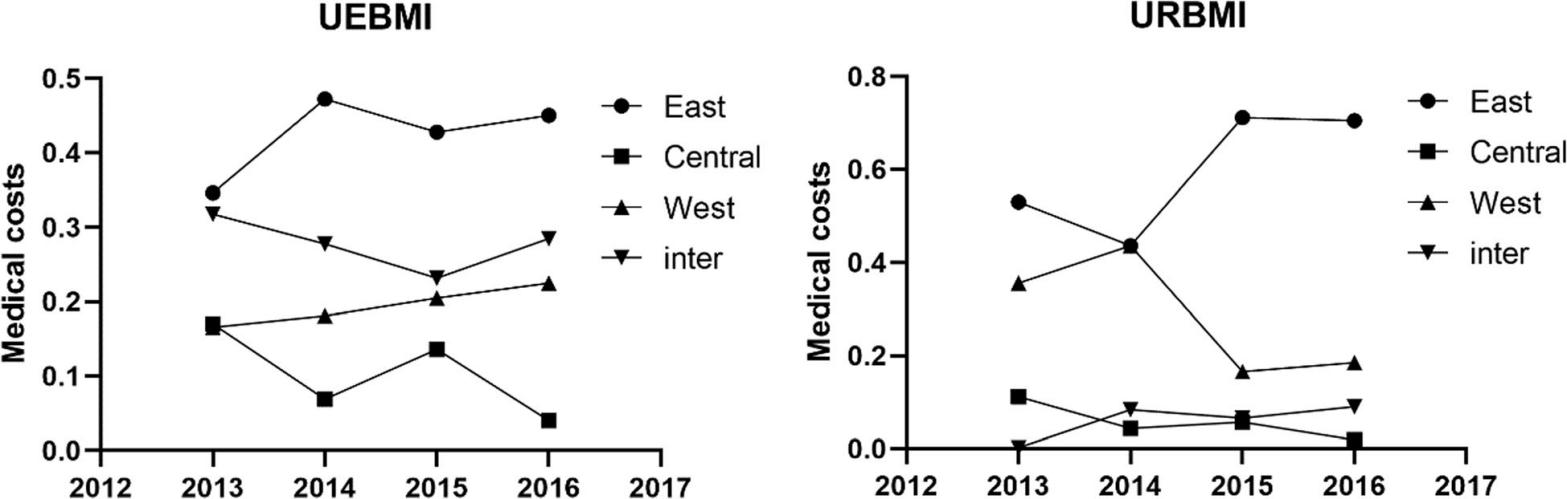
Theil index of medical costs from 2013 to 2016. Four curves in each subfigure denote the Theil index values within eastern, central and western China, and Theil index values between the three regions. X-axis represents the year and Y-axis denotes the Theil index value which was calculated by the formula (2) and (3) mentioned above. The right-side denotes the URBMI group and the left-side denotes Theil index values of the UEBMI group

**Fig. 2:**
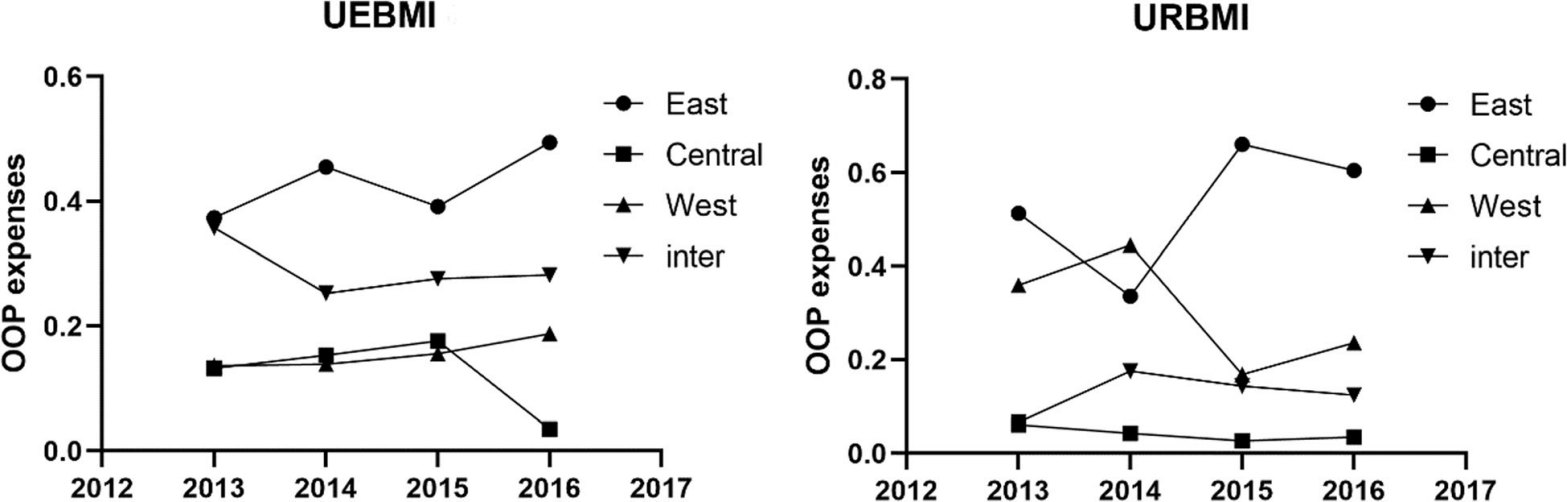
Theil index of OOP expenses from 2013 to 2016. Four curves in each subfigure denote the Theil index values within eastern, central and western China, and Theil index values between the three regions. X-axis represents the year and Y-axis denotes the Theil index value which was calculated by the formula (2) and (3) mentioned above. The right-side denotes the URBMI group and the left-side denotes Theil index values of the UEBMI group

**Fig. 3:**
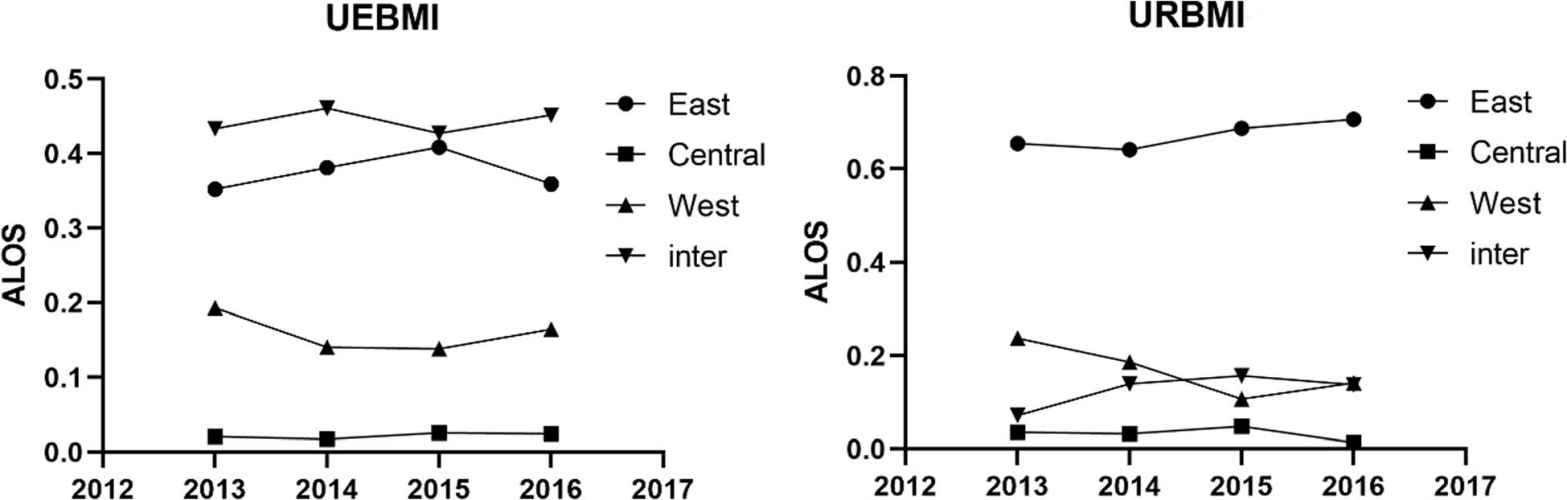
Theil index of ALOS from 2013 to 2016. Four curves in each subfigure denote the Theil index values within eastern, central and western China, and Theil index values between the three regions. X-axis represents the year and Y-axis denotes the Theil index value which was calculated by the formula (2) and (3) mentioned above. The right-side denotes the URBMI group and the left-side denotes Theil index values of the UEBMI group

An alternative illustration of inequalities in expenses and utilization is shown in Figs. 1, 2 and 3, which plots for each region the year-by-year Theil index for URBMI and UEBMI expenses and utilization. In the UEBMI and URBMI group, the eastern region had the highest Theil index of medical costs, OOP expenses, and ALOS over the four years, and the highest Theil index values between regions. The central and western region Theil index values were broadly similar, but displayed some, mainly small, different year-by-year fluctuations. The inter-region inequality indexs in Figs. 1, 2 and 3 show significant year-by-year fluctuations for UEBMI expenses and utilization, with the inter-region URBMI showing a more dampened year-by-year fluctuations in expenses and utilization. There were no significant trends in Figs. 1, 2 and 3 towards greater equality of hospital expenses and utilization within regions, between regions and between UEBMI and URBMI.

### Factors associated with medical costs and healthcare utilization

The random-effect panel model explored the provincial level factors influencing the geographical variation of stroke patients’ healthcare utilization and expenditure. We found that the health resource factors and enabling factors (average schooling year and regional reimbursement rate) had significant influence on patients’ medical costs and OOP expenses, while the impact effect varied by insurance type. As shown in Table 3, for medical cost, the coefficient of number of actual open beds per 1000 residents was − 0.089 (*P* < 0.001) in the UEBMI group, while it was − 0.128 (*P* < 0.001) in the URBMI group, which means number of hospital beds had significant negative impact on stroke patients’ medical costs and URBMI group was more sensitive to the number of hospital beds. The coefficients of number of healthcare staff per 1000 residents was 0.087 (*P* < 0.001) in the UEBMI group while it was 0.113 (*P* < 0.001) in the URBMI group, which means the number of healthcare staff was positively associated with medical cost. In terms of enabling factors, if the average schooling year increases by 1 year, medical costs in the UEBMI group will decrease by 12.9%, and will decrease by 18.3%, in the URBMI group. The reimbursement rate was negatively (Coef. = − 0.009, *P* = 0.004) associated with medical costs in URBMI group. For OOP expenses, the number of health care staff and GDP per capita had positive influence on OOP expenses both in UEBMI and URBMI group. While the number of hospital beds, average schooling year and reimbursement rate all were negatively associated with OOP expenses both in UEBMI and URBMI group (*P* < 0.05). However, factors except fund per capita were all identified no significant on stroke patients’ healthcare utilization.

**Table 3:** Province-level factors associated with stroke inpatients’ medical costs and healthcare utilization.

Variables	UEBMI			URBMI		
	Coef.	***P***-value	95% CI	Coef.	***P***-value	95% CI
**Medical costs**						
Beds	− 0.089	< 0.001	[− 0.134, − 0.043]	− 0.128	< 0.001	[− 0.192, − 0.064]
Staff	0.087	< 0.001	[0.040, 0.135]	0.113	< 0.001	[0.061, 0.165]
Education attainment years	−0.129	< 0.001	[− 0.197, − 0.062]	− 0.183	0.002	[− 0.300, − 0.065]
Urban employment rate	− 0.010	0.145	[− 0.023, 0.003]	0.010	0.442	[− 0.016, 0.037]
Reimbursement rate	0.003	0.406	[− 0.004, 0.010]	− 0.009	0.004	[− 0.016, − 0.003]
Fund per capita	5.55e^− 6^	0.293	[−1.59e^− 5^,4.80e^− 6^]	− 2.91e^− 6^	0.188	[− 7.25e^− 6^, − 1.43e^− 6^]
GDP per capita	8.50e^− 6^	0.001	[3.33e^− 6^,1.37e^− 5^]	5.89e^− 6^	0.122	[− 1.38e^− 6^,1.32e^− 6^]
**OOP expense**						
Beds	−0.099	< 0.001	[− 0.152, − 0.046]	−0.115	0.005	[−0.196, − 0.034]
Staff	0.101	< 0.001	[0.053, 0.149]	0.103	0.002	[0.037, 0.168]
Education attainment years	−0.108	0.002	[−0.177, − 0.039]	−0.183	0.001	[−0.293, − 0.073]
Urban employment rate	− 0.019	0.010	[− 0.034, − 0.005]	0.004	0.724	[− 0.017, 0.025]
Reimbursement rate	− 0.044	< 0.001	[− 0.055, − 0.033]	−0.025	< 0.001	[− 0.036, − 0.014]
Fund per capita	3.19e^−6^	0.479	[−1.20e^− 5^,5.63e^− 6^]	−4.18e^− 6^	0.091	[−9.03e^− 6^, 6.67e^− 6^]
GDP per capita	9.32e^− 6^	< 0.001	[4.76e^−6^,1.39e^− 5^]	7.49e^− 6^	0.042	[2.55e^− 7^,1.47e^− 5^]
**ALOS**						
Beds	−0.022	0.312	[−0.066,0.021]	− 0.026	0.301	[− 0.076, 0.024]
Staff	0.030	0.130	[−0.009,0.069]	0.031	0.332	[−0.032, 0.095]
Education attainment years	−0.049	0.113	[−0.110,0.112]	0.004	0.938	[−0.099, 0.108]
Urban employment rate	0.004	0.541	[−0.009,0.018]	0.018	0.096	[−0.003,0.040]
Reimbursement rate	0.005	0.243	[−0.003,0.012]	−0.002	0.119	[−0.005,0.001]
Fund per capita	−6.58e^−6^	0.012	[−1.17e^−5^, −1.45e^− 6^]	3.20e^**−** 6^	0.010	[− 5.61e^− 6^, −7.80e^− 6^]
GDP per capita	3.40e^−6^	0.119	[−8.71e^− 7^, 7.67e^− 6^]	−2.36e^− 6^	0.325	[− 7.07e^− 6^, 2.34e^− 6^]

UEBMI Urban Employee Basic Medical Insurance scheme, URBMI Urban Resident Basic Medical Insurance, Beds actual open beds per 1000 residents, Staff the number of healthcare staff per 1000 residents

## Discussion

For urban insurance schemes, this study provides a comprehensive nationwide exploration of equity in stroke patients’ healthcare utilization and expenses. Using the Theil index to measure “horizontal equity”, we found significant unfairness in stroke patients’ healthcare utilization and hospital expenses. The Theil index values in our study were significantly higher than Theil index values of government health care expenditure from 2013 to 2016, which were reported to range from 0.020 to 0.021(3). Theil index of health expenditure per capita in China, which ranged from 0.0583 to 0.0686 between 2013 and 2016 [29], also lower than Theil index value of stroke patients’ medical costs and healthcare utilization.

### Inequity in stroke patients in regard to healthcare utilization kept growing

A steady rise in the Theil index of ALOS both in UEBMI and URBMI group, indicated the growing inequity in ALOS. For all patients in China, Xie et al. [30] also found that the overall equity in patients in regard to healthcare utilization declined from 2011 to 2015. In general, declining equity in UEBMI and URBMI ALOS might be attributable to economy development gap and local hospitals administration disparities, etc. Since ALOS was one of the important performance assessment indexes, public hospitals in China actively reduced patients’ ALOS, by encouraging, for example, patients to be discharged early, to improve their performance [31]. Previous studies reported that multimorbidity, specifically hypertension, was a strong predictor of longer ALOS for stroke patients [32]. There were considerable geographic variations in prevalence of hypertension in China, and high hypertension prevalence zones, which extended from parts of the southeast to the northern and northeast [33, 34]. These different geographical hypertension prevalence zones would impact the geographical utilization rates of UEBMI and URBMI in Table 1. We also expect that difference of regional stroke prevention and treatment systems was an important factor leading to the geographic variation in patients’ ALOS. Under a well-functioning stroke prevention and treatment system, stroke patients could receive more accessbile and higher quality treatment in high-level hospitals than other regions with relative weaker stroke prevention and treatment systems. Delayed medical checks and treatment, and the limited treatment capacity of a hospital, could dramatically contribute to longer ALOS for stroke patients [35, 36]. Previous studies noted the large geographic variation in stroke prevention and treatment system in China [1], which may explain some of the inequity in stroke patients in regard to healthcare utilization. We recommend that public hospitals in China establish an unified and more scientific assessment index system for stroke patients and improve their capacity for treating stroke patients. The government should further strengthen the stroke prevention and treatment system, especially in poor areas with diminished healthcare delivery.

### The UEBMI stroke patients enjoyed greater fairness in medical costs and healthcare utilization than the URBMI patients

In this study, we emphasized on comparing fairness of patients’ medical costs and healthcare utilization by different insurance schemes. Our empirical evidence clearly revealed that the UEBMI group had an overall smaller Theil index of medical costs, OOP expenses and ALOS than the URBMI group from 2013 to 2016. That means stroke patients with URBMI experienced greater inequity in healthcare utilization and expenditure than those with UEBMI. Examining the equity in patients in regard to health service utilization in different regions, Zhang [37] also found patients covered by UEBMI had greater equity in healthcare utilization than those covered by URBMI. The UEBMI scheme provided more generous benefits, more comprehensive service coverage, as well as stronger financial protection [14]. Since the UEBMI scheme provided stronger financial protection than the URBMI scheme, UEBMI patients would seek a more comprehensive treatment than the URBMI patients [38]. With lower levels of benefits and financial protection, the URBMI patients would economize on their level of health services utilization subject to their family’s financial status [38]. This would contribute to inequity in healthcare utilization and hospital expenses. Importantly, patients covered by UEBMI had stable incomes due to their employment status and usually had a better financial situation than URBMI unemployed, retirees, students and children inpatients. Without worrying about the occurrence of catastrophic health expenditure, patients in different regions would receive treatment as required, but the UEBMI patients could incur higher OOP expenses and longer hospital stays [39]. Furthermore, a large proportion of patients covered by the URBMI were unemployed and children, with lower education levels than the UEBMI patients. Education level was considered an important factor which would affect the ALOS for stroke inpatients [36]. There were significant regional variations in education level, with people in eastern China having the highest education level, followed by the central and western regions. These regional education level differences probably interacted with the type of insured patient to contribute to inequity in UEBMI-URBMI healthcare utilization and hospital expenses.

### Eastern China was the main source of inequity

Figures 1, 2 and 3 displays the inequity in healthcare utilization and medical costs. We found that the internal differences within regions were major factors contributing to inequity in stroke patients in regard to healthcare utilization and health service expenses. Internal differences in the eastern region accounted for the largest part of the Theil index. When the gap of socio-economic development level between the richer eastern region and poorer western and northern regions was significant, it seemed counter intuitive that the richest region had the greatest inequity in healthcare utilization and health service expenses. The coastal areas and strong economic zones in eastern China had advantages of export-linked and foreign investment industry and enhanced infrastructure, and benefited most from economic policy reforms that transformed China’s economy, but that would possibly widen the economic gap between different cities in eastern China. Differences among cities in the central and western provinces were relatively smaller than within the eastern region. Therefore, socio-economic factors may be an important reason for the greater healthcare inequity within the eastern region. Another possible reason for the inequalities within the eastern and between the eastern and other regions was healthcare resource allocation. Previous studies reported that the eastern region had been experiencing the worst equity in health resource allocation [40], which was reflected in inequities in healthcare delivery. We recommend that the government should not only make policies to improve the medical system in central and western regions, but also take the less developed provinces and prefectures in eastern China into account.

### The health resource factors and enabling factors had significant influence on stroke patients’ medical costs and OOP expenses

Our results demonstrated that the number of actual open beds per 1000 residents at the provincial level which measured health resource factor had a significant negative impact on the medical costs and OOP expenses both in the UEBMI and URBMI group. While the number of healthcare staff per 1000 residents was positively associated with medical costs and OOP expenses both in these two groups. Health resources are considered as one of the important indicators reflecting the capacity of the healthcare system, and can determine the access to health services from the supply side. Previous studies have identified a positive relationship between health care and health resources [41, 42]. We believe that more staff improved patient’s healthcare utilization and caused higher expenses, which can be explained by Physician-Induced demand theory. In terms of the negative association between beds and medical costs, theoretically, more beds could significantly reduce the average fixed cost of a bed in a hospital, as well improve the ability of a hospital to generate revenue. We speculate that hospitals with more beds were faced with lower financial risk, thus, induce demand was less likely to happen. In addition, under China’s medical system, hospitals with more beds generally had stronger medical technology, which contributed to fewer medical costs for patients [43]. Thus, both the UEBMI and URBMI patients had fewer medical costs and OOP expenditure when a province equipped with more beds.

The province-level reimbursement rate which measured enabling factors was negatively associated with OOP expense both in UEBMI and URBMI group, and also had a negative influence on medical costs in URBMI group. That was consistent with findings in prior studies [44]. Higher reimbursement rate means stronger financial protection for patients. When protected by a strong enough health insurance system, patients are given the capacity and chance to utilize equal medical services, especially for vulnerable groups. The urban employment rate, which measured enabling factor, had a positive influence on medical costs in the URBMI group. Higher urban employment rate means more urban families had stable income, who are more likely to utilize health services and had higher medical costs, but less likely to incur catastrophic health expenditure [45]. Similarly, Lee’s study revealed that due to lack of proper financial safety net, patients could reduce healthcare utilization and non-adherence to treatment because of unemployment [46]. In terms of the negative impact of provincial average schooling years on medical costs and OOP expenses both in the UEBMI and URBMI group, there were several possible reasons behind that. First, with the growth of education attainment years, people’s awareness of maintaining physical and mental health has critically improved, thus people were more likely to take health-related behavior such as smoking cessation and keeping exercise. People with healthy bodies were considered easier to recover from illness and had fewer medical costs [47]. Second, with better education attainment, we speculate that people knew more about the National Health Insurance Directory, which endowed them with stronger ability to identify and refuse parts of health services uncovered in the Directory, and utilize more services covered in the Directory (costs of health services covered in the Directory will be compensated by insurance scheme) and causing fewer OOP expense. Third, better education attainment could possibly make people less likely to fall into the trap of induced demand of hospitals. And overtreatment due to demand side is also less likely to happen [48]. In addition, we found that the URBMI group was more sensitive to education attainment year than the UEBMI group when it comes to the impact on medical costs and OOP expenses. We speculate that people with UEBMI had an overall better education attainment than people with URBMI. Consequently, the marginal effect of education attainment year for the UEBMI group was weaker.

However, we found no statistical association between all indictors and healthcare utilization in this study. This may be because we selected only ALOS to represent stroke patients’ healthcare utilization at provincial level. Although we have known that health resource factors, enabling factors and economic factors have significant influence on healthcare utilization from literatures [25, 49], we didn’t have enough evidence to guarantee these indicators measuring province-level ALOS appropriately.

### Limitations

This study has a number of limitations. Firstly, our data applies to healthcare utilization and medical costs of urban stroke inpatients, findings might not be generalizable to the whole stroke patient population in China. Secondly, due to data limitations, we used ALOS to reflect healthcare utilization of stroke patients, but healthcare utilization based on need and demand cannot be easily divided. Therefore, the results should be interpreted with care. Thirdly, our data was from 2013 to 2016 which would be a little delayed. After 2016, URBMI has emerged with the new rural cooperative medical insurance (insurance mainly cover rural residents) which has greatly improved the equity between rural and urban aeras in China. Since gaps between URBMI and UEBMI still exists, we believe our research could contribute to further improvement on equity in stroke patients in regard to healthcare utilization.

## Conclusion

Under the fragmented health insurance system in China, there were significant disparities in healthcare utilization and medical costs within and between regions for stroke patients from the year 2013 to 2016. The internal differences in the eastern region which made the highest contribution to the Theil index were relatively larger than other regions. And the UEBMI group enjoyed greater equity in healthcare utilization and medical costs than the URBMI group. Health resources factors represented by the number of hospital beds per 1000 residents and the number of healthcare staff per 1000 residents, enabling factors represented by education attainment years and reimbursement rate, and economic factor represented by GDP per capita contributed to the identified unfairness of medical costs and OOP expenses. This study could be a model for future studies which intend to investigate fairness of costs for other illness interests. It is essential for policy makers to take effective measures to reduce inequity for stroke patients, such as consolidating the fragmented health insurance system at national level, providing equal benefit package and financial protection for both UEBMI and URBMI patients in different regions. Improving education attainment, offering equal access to healthcare, allocating health resources reasonably and balancing health services prices in different regions can also reduce patients’ medical costs and improve equity.

## Data Availability

The data that support the findings of this study are available from China Health Insurance Research Association, but restrictions apply to the availability of these data, which were used under license for the current study, and so are not publicly available. Data are however available from the authors upon reasonable request and with permission of China Health Insurance Research Association.

## Abbreviations

UEBMI: Urban Employee Basic Medical Insurance scheme
URBMI: Urban Resident Basic Medical Insurance scheme
OOP: out-of-pocket
ALOS: average length of stay
Beds: actual open beds per 1000 residents
Staff: the number of healthcare staff per 1000 residents
